# Journal of Veterinary Internal Medicine manuscript reviewers who critiqued in the 2023 calendar year

**DOI:** 10.1111/jvim.17014

**Published:** 2024-02-08

The Editorial Board thanks the following individuals for their time and effort spent in reviewing and critiquing manuscripts during the 2023 calendar year

Adams, Amanda; Kentucky, USA

Addie, Diane; France

Adin, Darcy; Florida, USA

Adkins, Pamela; Missouri, USA

Aherne, Michael; Florida, USA

Aicher, Kathleen; North Carolina, USA

Aleman, Monica; California, USA

Allenspach, Karin; Iowa, USA

Allerton, Fergus; United Kingdom of Great Britain and Northern Ireland

Al‐Nadaf, Sami; California, USA

Anderson, Katie; Wisconsin, USA

Andrews, Frank; Louisiana, USA

Armengou, Lara; Barcelona, Spain

Armstrong, Susan; United Kingdom of Great Britain and Northern Ireland

Arnold, Susan; Minnesota, USA

Aroch, Itamar; Jerusalem, Israel

Arroyo, Luis; Ontario, Canada

Atherton, Matthew; Pennsylvania, USA

Atiee, Genna; Texas, USA

Avery, Anne; Colorado, USA

Backus, Robert; Missouri, USA

Bagley, Rodney

Balcomb, Christie; New Zealand

Balkman, Cheryl

Bandt, Carsten; USA

Banzato, Tommaso; Italy

Baral, Randolph; New South Wales, Australia

Barber, Renee; Georgia, USA

Barker, Andrew; Ontario, Canada

Barker, Lucy; United Kingdom of Great Britain and Northern Ireland

Barko, Patrick; Illinois, USA

Baron Toaldo, Marco; Switzerland

Barrett, Renea; USA

Bartner, Lisa; Colorado, USA

Bass, Luke; Colorado, USA

Batchelor, Daniel; United Kingdom of Great Britain and Northern Ireland

Bauquier, Jennifer; Victoria, Australia

Baynes, Ronald; North Carolina, USA

Beasley, Michaela; Mississippi, USA

Beccati, Francesca; USA

Beck, Catherine; Victoria, Australia

Beckmann, Katrin; Zürich, Switzerland

Benchekroun, Ghita; Île‐de‐France, France

Bender, Susan; Pennsylvania, USA

Bennett, Peter; New South Wales, Australia

Berendt, Mette; Frederiksberg C, Denmark

Berger, Darren; USA

Berghoff, Nora; Michigan, USA

Berman, Julie; Quebec, Canada

Bernardini, Marco; Bologna, Italy

Bertin, Francois‐Rene; Queensland, Australia

Bertran, Judit; Florida, USA

Beukers, Martjin; The Netherlands

Bexfield, Nicholas; Cambridgeshire, United Kingdom of Great Britain and Northern Ireland

Bhatti, Sofie; Belgium

Bianchi, Matheus; Brazil

Bienzle, Dorothee; Ontario, Canada

Bierlein, Metzere; California, USA

Birettoni, Francesco; Italy

Bissett, Sally; North Carolina, USA

Blackwood, Laura; Cheshire West and Chester, United Kingdom of Great Britain and Northern Ireland

Blois, Shauna; Ontario, Canada

Blot, Stéphane; France

Boag, Alisdair; United Kingdom of Great Britain and Northern Ireland

Bode, Elizabeth; Cheshire, United Kingdom of Great Britain and Northern Ireland

Bojanic, Krunoslav; Croatia

Boland, Lara; New South Wales, Australia

Bonelli, Marilia; Ohio, USA

Boretti, Felicitas; Switzerland

Borgeat, Kieran; United Kingdom of Great Britain and Northern Ireland

Boswood, Adrian; Hertfordshire, United Kingdom of Great Britain and Northern Ireland

Boudreau, C. Elizabeth; Texas, USA

Boudreaux, Bonnie; USA

Bowal, Jacqueline; Ontario, Canada

Boyd, Corrin; Western Australia, Australia

Boyle, Ashley; Pennsylvania, USA

Bracha, Shay; USA

Bradford, Brad; USA

Brainard, Benjamin; Georgia, USA

Breheny, Craig; United Kingdom of Great Britain and Northern Ireland

Bristow, Poppy; United Kingdom of Great Britain and Northern Ireland

Brito‐Casillas, Yeray; Canarias, Spain

Broeckx, Bart; Belgium

Browne, Nimet; Kentucky, USA

Bryan, Jeffrey; Missouri, USA

Buczinski, Sebastien; Quebec, Canada

Buller, Maggie; California, USA

Bullone, Michela; TO, Italy

Burge, Rhonda; Ohio, USA

Burgess, Brandy; Georgia, USA

Buriko, Yekaterina

Burns, Teresa; Ohio, USA

Burton, Jenna; Colorado, USA

Busch, Kathrin; Germany

Busch, Rosie; California, USA

Busechian, Sara; Italy

Bush, William; Maryland, USA

Byrne, Barbara; California, USA

Byrne, David; Western Australia, Australia

Byron, Julie; Ohio, USA

Cameron, Starr; Wisconsin, USA

Cannon, Claire; Victoria, Australia

Cardy, Tom; United Kingdom of Great Britain and Northern Ireland

Carey, Stephan; Michigan, USA

Carr, Elizabeth; Michigan, USA

Carrera‐Justiz, Sheila; Florida, USA

Castillo, V. A.; Argentina

Catalfamo, Jim; New York, USA

Cavanaugh, Sarah; Saint Kitts and Nevis

Ceplecha, Vaclav; Czech Republic

Chacar, Fernanda; Sao Paulo, Brazil

Chako, Clemence; Arizona, USA

Chalhoub, Serge; Alberta, Canada

Chamorro, Manuel; Alabama, USA

Charalambous, Marios; Germany

Chen, Hilla; Not Applicable, Israel

Chew, Dennis; Ohio, USA

Chidlow, Hayley; Georgia, USA

Childress, Michael; Indiana, USA

Choi, Eunju; California, USA

Chon, Esther; Wisconsin, USA

Christmann, Undine; Tennessee, USA

Christopherson, Peter; Alabama, USA

Churchill, Julie; Minnesota, USA

Clark, Mitzi USA

Clarke, Dana; Pennsylvania, USA

Clergue, Suzanne; France

Clifford, Craig; Pennsylvania, USA

Coelho, Ana Maria; USA

Coffey, Emily; Minnesota, USA

Coggins, Sally; New South Wales, Australia

Coleman, Amanda; Georgia, USA

Contreras, Elena; New York, USA

Conway, Elizabeth; United Kingdom of Great Britain and Northern Ireland

Cook, Laurie; Ohio, USA

Cook, Shawna; New York, USA

Cooke, Kirsten; Florida, USA

Cooper, Camilla; USA

Cooper, Jocelyn; Texas, USA

Corbee, Ronald; The Netherlands

Cordella, Alessia; Emilia‐Romagna, Italy

Cornelis, Ine; Belgium

Corsini, Andrea; Italy

Cosford, Kevin; Saskatchewan, Canada

Couetil, Laurent; Indiana, USA

Couto, Guillermo; Ohio, USA

Credille, Brent; Georgia, USA

Cribb, Alastair; Massachusetts, USA

Cridge, Harry; Michigan, USA

Crowe, Chelsea; California, USA

Cullen, John; North Carolina, USA

Cunningham, Suzanne; Massachusetts, USA

Cuq, Benoît; Ireland

Cvejic, Dejan; Germany

Czerwik, Adriana; Germany

da Costa, Ronaldo; Ohio, USA

Daminet, Sylvie; Belgium

Dandrieux, Julien; United Kingdom of Great Britain and Northern Ireland

Daniels, Joshua; Colorado, USA

Davis, Elizabeth; Kansas, USA

De Decker, Steven; Hertfordshire, United Kingdom of Great Britain and Northern Ireland

de Laat, Melody; Queensland, Australia

De Laforcade, Armelle; Massachusetts, USA

Dear, Jonathan; California, USA

Decloedt, Annelies; Belgium

Defauw, Pieter; Hampshire, United Kingdom of Great Britain and Northern Ireland

DeFrancesco, Teresa; North Carolina, USA

Del Baldo, Francesca; Bologna, Italy

Del Nero, Bruna; Florida, USA

Dembek, Katarzyna; North Carolina, USA

Denes, Bela; USA

Dennler, Matthias; Switzerland

Depenbrock, Sarah; California, USA

DeStefano, Ian; Massachusetts, USA

Deveau, Michael; Texas, USA

Dewell, Grant; USA

Diaz‐Campos, Dubraska; Ohio, USA

Dickinson, Peter; California, USA

DiFazio, Matthew; Kansas, USA

Dijk, Jan van; USA

Distl, Ottmar; Germany

Divers, Thomas; New York, USA

Djani, Dylan; South Carolina, USA

Donnelly, Callum; New York, USA

Dore, Vincent; Quebec, Canada

Driver, Colin J.; USA

Drögemüller, Cord; Switzerland

Dufayet, Cedric; California, USA

Dunkel, Bettina; Herts, United Kingdom of Great Britain and Northern Ireland

Dunn, Marilyn; Quebec, Canada

Durward‐Akhurst, Sian; Minnesota, USA

Dutrow, Emily; New Jersey, USA

Dutton, Emily; Cheshire, United Kingdom of Great Britain and Northern Ireland

Duval, Dawn

Dyrka, Magdalena; Glasgow, United Kingdom of Great Britain and Northern Ireland

Ekenstedt, Kari; Indiana, USA

ElAshmawy, Wagdy; California, USA

Elliott, James; United Kingdom of Great Britain and Northern Ireland

Elliott, Jonathan; United Kingdom of Great Britain and Northern Ireland

Estell, Krista; Virginia, USA

Evans, Samantha; Ohio, USA

Fadda, Angela; Avon, United Kingdom of Great Britain and Northern Ireland

Faller, Kiterie; United Kingdom of Great Britain and Northern Ireland

Fecteau, Marie‐Eve; Pennsylvania, USA

Feijoó, Silvia; Argentina

Feldman, Edward; California, USA

Fellman, Claire; Massachusetts, USA

Fenn, Joe; Hertfordshire, United Kingdom of Great Britain and Northern Ireland

Ferraro, Salvatore; Uppsala, Sweden

Ferri, Filippo; Novara, Italy

Ferrucci, Francesco; Italy

Fidel, Janean; Washington, USA

Fink‐Gremmels, Johanna; The Netherlands

Finnen, Andrea; Ontario, Canada

Finno, Carrie; California, USA

Finotello, Riccardo; Italy

Fischer, Andrea; Bayern, Germany

Flegel, Thomas; Germany

Fonseca‐Alves, Carlos Eduardo; USA

Foss, Kari; Illinois, USA

Foster, Jonathan; District of Columbia United States

Foster, Robert; Ontario, Canada

Fox, Philip; New York, USA

Foy, Daniel; Arizona, USA

Fracassi, Federico; Italy

Francey, Thierry; Switzerland

Franklin, Samantha; South Australia, Australia

Freeman, Lisa; Massachusetts, USA

Friedenberg, Steven; Minnesota, USA

Fries, Ryan; Illinois, USA

Fritz, Heather; California, USA

Fulkerson, Christopher; Indiana, USA

Furr, Martin; Oklahoma, USA

Gagnon, Allison; California, USA

Galvao, Felipe; Illinois, USA

Gamsjäger, Lisa; Alberta, Canada

García San José, Paula; Madrid, Spain

Garcia Sancho, Mercedes; Madrid, Spain

Garcia, Pablo; Madrid, Spain

Gaschen, Frederic; Louisiana, USA

Geddes, Rebecca; United Kingdom of Great Britain and Northern Ireland

Gedye, Kristene; New Zealand

Gentile, Arcangelo; Italy

Gerber, Vinzenz; Switzerland

Ghantous, Seth; Florida, USA

Giannuzzi, Diana; Italy

Gieger, Tracy; North Carolina, USA

Gilbertie, Jessica; Virginia, USA

Gilkerson, James; Victoria, Australia

Gilor, Chen; Florida, USA

Giorgi, Mario; Pisa, Italy

Glaus, Tony; Switzerland

Glynn, Sarah; Ad‐Dawhah, Qatar

Gnirs, Kirsten; France

Goddard, Amelia; Gauteng, South Africa

Goggs, Robert; New York, USA

Gold, Jenifer; Washington, USA

Gomes, Sergio Andrade; Derbyshire, United Kingdom of Great Britain and Northern Ireland

Gomez, Diego; Ontario, Canada

Goncalves, Rita; United Kingdom of Great Britain and Northern Ireland

Goodman, Laura; USA

Gookin, Jody; North Carolina, USA

Gordin, Emilia; Finland

Gordon, Sonya; Texas, USA

Gori, Eleonora; Italy

Gostelow, Ruth; Hertfordshire, United Kingdom of Great Britain and Northern Ireland

Gould, Emily; Texas, USA

Graham, Joanne; Missouri, USA

Granat, Fanny; USA

Granger, Nicolas; Bristol, United Kingdom of Great Britain and Northern Ireland

Granick, Jennifer; Minnesota, USA

Grant, David; Virginia, USA

Grapes, Nick; United Kingdom of Great Britain and Northern Ireland

Greensmith, Tom; United Kingdom of Great Britain and Northern Ireland

Greer, Kimberly; Indiana, USA

Grissett, Gretchen; Mississippi, USA

Grobman, Megan; Alabama, USA

Guardabassi, Luca; Denmark

Guess, Sarah; Washington, USA

Guglielmini, Carlo; Italy

Gull, Tamara; Missouri, USA

Gutierrez‐Quintana, Rodrigo; United Kingdom of Great Britain and Northern Ireland

Häggström, Jens; Sweden

Hague, Devon; Illinois, USA

Hahn, Caroline; Scotland, United Kingdom of Great Britain and Northern Ireland

Hale, Anne; New Mexico, USA

Hall, Alison; United Kingdom of Great Britain and Northern Ireland

Hall, Jean; Oregon, USA

Hansen, Bernie; North Carolina, USA

Harcourt‐Brown, Thomas; Avon, United Kingdom of Great Britain and Northern Ireland

Harkin, Kenneth; Kansas, USA

Harris, Autumn; Florida, USA

Harris, Georgina; Cambridgeshire, United Kingdom of Great Britain and Northern Ireland

Hasegawa, Daisuke; Tokyo, Japan

Haspeslagh, Maarten; Belgium

Hawkins, Eleanor; North Carolina, USA

Hay, Alayna; Virginia, USA

Hecht, Silke

Heilmann, Romy; Saxony, Germany

Heller, Meera; California, USA

Henneman, Kimberly; Utah, USA

Hepburn, Richard; Gloucestershire, United Kingdom of Great Britain and Northern Ireland

Herstad, Kristin; Norway

Hess, Rebecka; Pennsylvania, USA

Hewetson, Michael; Hertfordshire, United Kingdom of Great Britain and Northern Ireland

Hezzell, Melanie; United Kingdom of Great Britain and Northern Ireland

Hill, Jonathan; New South Wales, Australia

Hill, Richard; Florida, USA

Hill, Tracy; Minnesota, USA

Holm, Sarah; New York, USA

Holt, Natalee; Maine, USA

Hooijberg, Emma; South Africa

Hopster‐Iversen, Charlotte; Denmark

Hostnik, Eric; Ohio, USA

House, John; New South Wales, Australia

Hsieh, Emmelyn; Pennsylvania, USA

Hsue, Weihow; New York, USA

Hulsebosch, Sean; California, USA

Hume, Kelly; New York, USA

Humm, Karen; United Kingdom of Great Britain and Northern Ireland

Husnik, Roman; Tennessee, USA

Ivester, Kathleen; Indiana, USA

Jablonski, Sara; Michigan, USA

Jackson, Wendi; California, USA

Jacob, Megan, USA

Jager, Mason; New York, USA

James, Fiona; Ontario, Canada

Jandrey, Karl; California, USA

Janes, Jennifer; Kentucky, USA

Jeffery, Nicholas; Texas, USA

Jepson, Rosanne; Herts, United Kingdom of Great Britain and Northern Ireland

Jessen, Lisbeth; Denmark

Johnson, Amy; Pennsylvania, USA

Johnson, Gary; Missouri, USA

Johnson, Lynelle; California, USA

Johnson, Tyler; North Carolina, USA

Johnston, Andrea; Louisiana, USA

Jokinen‐Pääkkönen, Tarja; Finland

Jones, Bethan; United Kingdom of Great Britain and Northern Ireland

Jones, Meredyth; Oklahoma, USA

José‐López, Roberto; United Kingdom of Great Britain and Northern Ireland

Jugan, Maria; Kansas, USA

Jukier, Tom; Alabama, USA

Jurina, Konrad; Germany

Kanazono, Shinichi; Saitama, Japan

Kang, Heng; Canada

Kaplan, Joanna; California, USA

Kather, Stefanie; Germany

Kathrani, Aarti; United Kingdom of Great Britain and Northern Ireland

Katz, Martin; Missouri, USA

Kedrowicz, April; North Carolina, USA

Keene, Bruce; North Carolina, USA

Kelley, Ashley; Florida, USA

Kellihan, Heidi; Wisconsin, USA

Kelly, Darren; Hampshire, United Kingdom of Great Britain and Northern Ireland

Kent, Michael; California, USA

Kidd, Linda; California, USA

Kim, Jon; Florida, USA

Kim, Sangho; Japan

King, Ailbhe; North Carolina, USA

Kirschvink, Nathalie; Belgium

Kisseberth, William

Klever, Julius; United Kingdom of Great Britain and Northern Ireland

Klohnen, Andreas; California, USA

Knowles, Edward, USA

Knych, Heather; California, USA

Kooistra, Hans; The Netherlands

Kook, Peter; Zurich, Switzerland

Kopecny, Lucy; California, USA

Kopper, Jamie; Washington, USA

Kornegay, Joe; Texas, USA

Köster, Liza; Tennessee, USA

Kraemer, Anna Lena; United Kingdom of Great Britain and Northern Ireland

Kreuder, Amanda; Iowa, USA

Krogh, Anne, USA

Kromhout, Kaatje; Belgium

Kruckman, Leah, USA

Kruitwagen, Hedwig; The Netherlands

KuKanich, Butch; Kansas, USA

KuKanich, Kate; Kansas, USA

Kuzi, Sharon; Israel

Labato, Mary Anna; Massachusetts, USA

Lacorcia, Lauren; United Kingdom of Great Britain and Northern Ireland

Ladlow, Jane; United Kingdom of Great Britain and Northern Ireland

Laflamme, Dorothy; Virginia, USA

Lana, Susan; Colorado, USA

Langlois, Daniel; Michigan, USA

Langston, Cathy; Ohio, USA

Lanier, Christopher; Florida, USA

Lantz, Daniel; New Jersey, USA

Larsen, Jennifer; California, USA

Lascelles, Duncan; North Carolina, USA

Lassaline, Mary; Pennsylvania, USA

Lathan, Patty; Louisiana, USA

Laursen, Sigrid; Denmark

Laver, Travis; Georgia, USA

Lawson, April; United Kingdom of Great Britain and Northern Ireland

Lawson, Jack; United Kingdom of Great Britain and Northern Ireland

Le Roux, Delphine, USA

Le Sueur, Andre; Sao Paulo, Brazil

Leal, Rodolfo; Portugal

Leclere, Mathilde; Quebec, Canada

Lee, Jih‐Jong; Taiwan

Lee, Robert, USA

Lee, Ya‐Jane; Taiwan

Leeb, Tosso; Switzerland

Leeder, Stephanie; New South Wales, Australia

Lee‐Fowler, Tekla; Alabama, USA

Leela‐arporn, Rommaneeya; Hokkaido, Japan

Leguillette, Renaud; Alberta, Canada

Leisewitz, Andrew; Alabama, USA

Lennon, Elizabeth; Pennsylvania, USA

Lenz, Jennifer; Pennsylvania, USA

Letko, Anna; Switzerland

Lewis, Melissa; Indiana, USA

Li, Ronald; North Carolina, USA

Lidbury, Jonathan; Texas, USA

Lilliehöök, Inger; Sweden

Lin, Chung‐Hui; Taiwan

Lindner, Arno

Lisciandro, Gregory; Texas, USA

Ljungvall, Ingrid; Sweden

Llobat, Lola; Spain

Lobetti, Remo; South Africa

Loeber, Samantha

Loftus, John; New York, USA

Logan, Darren, USA

Lohi, Hannes; Finland

Long, Sam; Victoria, Australia

Lorenz, Ingrid; Germany

Lourenço, Bianca; Georgia, USA

Lowrie, Mark; Derbyshire, United Kingdom of Great Britain and Northern Ireland

Lucidi, Cynthia; Michigan, USA

Luethy, Daniela; Pennsylvania, USA

Lulich, Jody; Minnesota, USA

Lunn, Katharine; North Carolina, USA

Lunn, Paul; United Kingdom of Great Britain and Northern Ireland

Lurie, David; New South Wales, Australia

Lynch, Alex; North Carolina, USA

Lyon, Shane; Kansas, USA

Lyu, Yang; East flanders, Belgium

Ma, Yunzhou; California, USA

MacKay, Robert; Florida, USA

Mackenzie, Catriona; USA

Macon, Erica; Kentucky, USA

Maddison, Jill; Herfordshire, United Kingdom of Great Britain and Northern Ireland

Madigan, John; California, USA

Mahony, Orla; Massachusetts, USA

Maier, Gabriele; California, USA

Maitz, Charles; Missouri, USA

Makielski, Kelly; Minnesota, USA

Malalana, Fernando

Malik, Richard; New South Wales, Australia

Manchester, Alison; Colorado, USA

Mandigers, Paul; The Netherlands

Manens, Jefferson; British Columbia, Canada

Mansfield, Caroline; Victoria, Australia

Marenzoni, Maria Luisa; USA

Marioni‐Henry, Katia; Midlothian, United Kingdom of Great Britain and Northern Ireland

Markovic, Lauren; Georgia, USA

Marolf, Angela; Ohio, USA

Matiasek, Kaspar; Bavaria, Germany

Matiasek, Lara; Germany

Matsuyama, Arata; Ontario, Canada

Maunder, Christina; United Kingdom of Great Britain and Northern Ireland

Maunsell, Fiona; Florida, USA

Mavangira, Vengai; Iowa, USA

Maxwell, Elizabeth; Florida, USA

Maxwell, Lara

Mayhew, Joe; New Zealand

Mazan, Melissal Massachusetts, USA

McCallum, Katie; United Kingdom of Great Britain and Northern Ireland

McElroy, Abigaill Massachusetts, USA

McKeever, Kenneth; New Jersey, USA

McLeland, Shannon; Ohio, USA

McMichael, Maureen; Alabama, USA

McNabb, Bret; California, USA

Meier, Valeria; Zurich, Switzerland

Meij, Bjorn; The Netherlands

Mellor, Paul, USA

Menciotti, Giulio; Virginia, USA

Menzies‐Gow, Nicola; Herts, United Kingdom of Great Britain and Northern Ireland

Merbl, Yael; New York, USA

Merhof, Kristina; Niedersachsen, Germany

Messam, Locksley, USA

Meyer, Allison; Missouri, USA

Meyer, James; New York, USA

Meyerhoff, Nina, Lower Saxony, Germany

Miller, Andrew; New York, USA

Milner, Rowan; Florida, USA

Miranda, Marta; Lugo, Spain

Mitchell, Katharyn; New York, USA

Mizuno, Takuya; Japan

Mochel, Jonathan; Iowa, USA

Moise, N. Sydney; New York, USA

Montgomery, Julia; Saskatchewan, Canada

Mooney, Carmel; Ireland

Moore, George; Indiana, USA

Moore, Sarah; Ohio, USA

Moore, Talisha; Tennessee, USA

Moreira da Silva, Joana; Portugal

Morresey, Peter; Kentucky, USA

Muñana, Karen; North Carolina, USA

Murakami, Keiko; Iowa, USA

Murphy, Maryanne; Tennessee, USA

Murthy, Vishal; California, USA

Nabity, Mary; Texas, USA

Nafe, Laura; Missouri, USA

Nagy, Dusty; Texas, USA

Nauwynck, Hans, USA

Negrin, Arianna; Spain

Nemec Svete, Alenka; Slovenia

Neves, Raphael, USA

Nicholas, Frank, USA

Nickel, Rafael; Germany

Niessen, Stijn; Herts, United Kingdom of Great Britain and Northern Ireland

Nishida, Hidetaka; Japan

Nissen, Sarah; Denmark

Norton, Elaine; Arizona, USA

Nostell, Katarina; Sweden

Nout‐Lomas, Yvette; Colorado, USA

Oberbauer, A. M.; California, USA

Oetzel, Garrett R.

Oevermann, Anna; Switzerland

Offer, Katie; United Kingdom of Great Britain and Northern Ireland

Ojeda, Javier; Chile

O'Kell, Allison; California, USA

Olby, Natasha; North Carolina, USA

Oliveira‐Filho, José; Sao Paulo, Brazil

Olsen, Lisbeth Høier; Denmark

Oman, Rachel; Colorado, USA

O'Neill, Emma; Dublin, Ireland

O'Sullivan, Lynne; Prince Edward Island, Canada

Otte, Corma; Utrecht, The Netherlands

Outerbridge, Catherine; California, USA

Overall, Karen; Prince Edward Island, Canada

Owens, Tammy; Saskatchewan, Canada

Oyama, Mark; Pennsylvania, USA

Packer, Rowena; United Kingdom of Great Britain and Northern Ireland

Paepe, Dominique; Belgium

Paillot, Romain; United Kingdom of Great Britain and Northern Ireland

Pakozdy, Akos; Austria

Palerme, Jean‐Sébastien; Iowa, USA

Pankraz, Alexander; Rheinland‐Pfalz, Germany

Papich, Mark; North Carolina, USA

Parker, Valerie; Ohio, USA

Parmentier, Thomas; Quebec, Canada

Parys, Maciej; United Kingdom of Great Britain and Northern Ireland

Patel, Reema; Virginia, USA

Patterson, Edward; Minnesota, USA

Paulussen, Ellen; Flanders, Belgium

Peaston, Anne; South Australia, Australia

Pellin, MacKenzie; Wisconsin, USA

Penning, Louis; Utrecht, The Netherlands

Pesato, Michael; Delaware, USA

Peters, Rosanne; Illinois, USA

Peterson, Mark; New York, USA

Petri, Renee; Quebec, Canada

Phillipps, Stephanie; Cheshire West and Chester, United Kingdom of Great Britain and Northern Ireland

Piercy, Richard; United Kingdom of Great Britain and Northern Ireland

Pihl, Tina; Denmark

Pilla, Rachel; Texas, USA

Pinello, Katia; Portugal

Piotrowski, Isabelle; Switzerland

Plonek, Marta; Poland

Pluhar, G. Elizabeth; Minnesota, USA

Pollard, Rachel; California, USA

Pöppl, Álan; Rio Grande do Sul, Brazil

Pozzato, Nicola, USA

Priestnall, Simon; Hatfield, United Kingdom of Great Britain and Northern Ireland

Prieto, Jennifer; New York, USA

Prudent, Michel; Switzerland

Puggioni, Antonella; Ireland

Quimby, Jessica; Ohio, USA

Raabis, Sarah; Colorado, USA

Rademacher, Nathalie; Louisiana, USA

Raheb, Shari; Ontario, Canada

Raidal, Sharanne; New South Wales, Australia

Raimondi, Francesca; Hampshire, United Kingdom of Great Britain and Northern Ireland

Rajamäki, Minna; Finland

Ramsey, Ian; United Kingdom of Great Britain and Northern Ireland

Rand, J. S.; Queensland, Australia

Reagan, Krystle; California, USA

Reed, Shannon; Texas, USA

Reed, Stephen; Kentucky, USA

Reetz, Jennifer; Pennsylvania, USA

Refsal, Kent; Michigan, USA

Reinhart, Jennifer; Illinois, USA

Richard, Eric; France

Ridyard, Alison; United Kingdom of Great Britain and Northern Ireland

Rigas, Konstantinos; United Kingdom of Great Britain and Northern Ireland

Riggers, Denise; Germany

Riihimaki, Miia; Sweden

Rings, Lindsey; Ohio, USA

Rishniw, Mark; New York, USA

Rivas, Victor North Carolina, USA

Robin, M.; United Kingdom of Great Britain and Northern Ireland

Roche‐Catholy, Marine; Belgium

Rohdin, Cecilia; Sweden

Romito, Giovanni; Bologna, Italy

Rosati, Marco; Germany

Roscher, Katja; Germany

Rossmeisl, John; Virginia, USA

Rotter, Carina; United Kingdom of Great Britain and Northern Ireland

Roura, Xavier

Royaux, Emilie; United Kingdom of Great Britain and Northern Ireland

Rozanski, Elizabeth; Massachusetts, USA

Ruaux, Craig; New South Wales, Australia

Rylander, Helena; Wisconsin, USA

Saba, Corey; Georgia, USA

Sabatino, Bethany; Tennessee, USA

Saellstrom, Sara, USA

Sanchez‐Masian, Daniel; United Kingdom of Great Britain and Northern Ireland

Sanderson, Sherry; Georgia, USA

Santangelo, Kelly, USA

Santos, Andrea; Indiana, USA

Scalco, Rebeca; California, USA

Schaefer, Gabriela; Rio Grande do Sul, Brazil

Schermerhorn, Thomas; Kansas, USA

Schmid, Sarah; Tennessee, USA

Schoeman, Johan; Gauteng, South Africa

Schoster, Angelika; Germany

Schott II, Harold; Michigan, USA

Schukken, Ynte, USA

Schulz, Bianka; Germany

Schwarz, Tobias; United Kingdom of Great Britain and Northern Ireland

Schweizer, Daniela; Bern, Switzerland

Scott‐Moncrieff, Catharine; Indiana, USA

Scudder, Christopher; Hertfordshire, United Kingdom of Great Britain and Northern Ireland

Seage, Eileen, USA

Segev, Gilad; Israel

Seibert, Rachel, USA

Sellon, Rance; Washington, USA

Selting, Kim; Illinois, USA

Seo, Joonbum; New Zealand

Serrano, Goncalo; Belgium

Seth, Mayank; United Kingdom of Great Britain and Northern Ireland

Shamoun, John; North Carolina, USA

Sharp, Claire; Western Australia, Australia

Sharpe, Ashley; North Carolina, USA

Sheats, Mary; North Carolina, USA

Shelton, G. Diane; California, USA

Shepherd, Megan; Virginia, USA

Sherding, Robert; Ohio, USA

Shihab, Nadia, USA

Shopshire, Sarah, USA

Shores, Andy; Mississippi, USA

Shropshire, Sarah; Colorado, USA

Silverstein, Deborah; Pennsylvania, USA

Silvestrini, Paolo; Pennsylvania, USA

Simoes, Joana; Portugal

Simpson, Katharine; Colorado, USA

Simpson, Kathryn; Illinois, USA

Simpson, Kenneth; New York, USA

Singh, Mithilesh, USA

Skorupski, Katherine; California, USA

Slead, Tanner; North Carolina, USA

Slovak, Jennifer; Wyoming, USA

Smets, Pascale; Belgium

Sojka Kritchevsky, Janice; Indiana, USA

Solano, Mauricio; Massachusetts, USA

Soler Arias, Elber; Argentina

Spada, Eva; Italy

Spadavecchia, Claudia; Switzerland

Spalla, Ilaria; United Kingdom of Great Britain and Northern Ireland

Specht, Andrew; Florida, USA

Spillmann, Thomas; Finland

Stabile, Fabio; United Kingdom of Great Britain and Northern Ireland

Stauthammer, Christopher; Minnesota, USA

Stee, Kimberley; Belgium

Steenbrugge, Jonas, USA

Steffen, Frank; Zürich, Switzerland

Stein, Timothy; Texas, USA

Steiner, Joerg; Texas, USA

Stepien, Rebecca; Wisconsin, USA

Stevenson, Mark; Victoria, Australia

Stockman, Jonathan; New York, USA

Stone, Michael; Massachusetts, USA

Stowe, Devorah; North Carolina, USA

Strage, Emma; Sweden

Strain, George; Louisiana, USA

Suchodolski, Jan; Texas, USA

Sullivant, Alyssa; Mississippi, USA

Suter, Steven; North Carolina, USA

Sutherland, Katja; Ontario, Canada

Suzuki, Ryohei; Tokyo, Japan

Swiderski, Cyprianna; Arizona, USA

Sykes, Benjamin, USA

Sylvester, Skylar, USA

Syme, Harriet Herts; United Kingdom of Great Britain and Northern Ireland

Syrja, Pernilla; Finland

Taboada, Joseph; Louisiana, USA

Talcott, Patricia, USA

Tallon, Rose; United Kingdom of Great Britain and Northern Ireland

Tamborini, Alice; Ireland

Tamura, Masahiro; Japan

Tappin, Simon; Suffolk, United Kingdom of Great Britain and Northern Ireland

Tate, Nicole; Minnesota, USA

Tayler, Sarah; Hertfordshire, United Kingdom of Great Britain and Northern Ireland

Teske, Erik; The Netherlands

Thomas, William; Tennessee, USA

Thomason, John; Mississippi, USA

Thomovsky, Elizabeth; Indiana, USA

Thomovsky, Stephanie; Indiana, USA

Thompson, Craig; Indiana, USA

Thompson, Dan; United Kingdom of Great Britain and Northern Ireland

Tidholm, Anna; Sweden

Tolbert, M. Katherine; Texas, USA

Trefz, Florian; Germany

Trepanier, Lauren; Wisconsin, USA

Trewin, Isla; England, United Kingdom of Great Britain and Northern Ireland

Tyrrell, William; Virginia, USA

Tzounakas, Vassilis; Greece

Ueda, Yu; North Carolina, USA

Ullal, Tarini; California, USA

Underwood, Claire; Pennsylvania, USA

Valldecabres, Ainhoa; Ireland

Van Cleemput, Jolien; Belgium

van Dongen, Astrid; The Netherlands

van Eps, Andrew; Pennsylvania, USA

Van Erck‐Westergren, Emmanuelle; Belgium

van Maanen, Kees; The Netherlands

Van Schyndel, Sabrina; Ontario, Canada

Vandevelde, Marc; Switzerland

Vanhaesebrouck, An; Cambridgeshire, United Kingdom of Great Britain and Northern Ireland

VanHoy, Grace; California, USA

Vascellari, Marta; Padua, Italy

Vecchiato, Carla; Italy

Venn, Emilee; Maryland, USA

Vezzosi, Tommaso; Pisa, Italy

Viall, Austin; California, USA

Vickery, Kathryn; Colorado, USA

Viitanen, Sanna; Finland

Villm, Jessica; Colorado, USA

Viviano, Katrina; Wisconsin, USA

von Euler, Henrik, USA

von Rüden, Lotta; Germany

Wakshlag, Joseph; New York, USA

Waldridge, Bryan; Mississippi, USA

Walker, Julie; Wisconsin, USA

Wallace, Mandy

Wang, Xu; Alabama, USA

Wang‐Leandro, Adriano; Germany

Ward, Jessica; Iowa, USA

Warnken, Tobias; Lower Saxony, Germany

Washburn, Kevin; Texas, USA

Watson, Penny; United Kingdom of Great Britain and Northern Ireland

Weaver, Leslie; Kansas, USA

Webb, Craig; Colorado, USA

Webster, Cynthia; Massachusetts, USA

Weese, J. Scott; Ontario, Canada

Wehner, Astrid; Germany

Weisse, Chick; New York, USA

Werner, Melanie; Switzerland

Wessmann, Annette; United Kingdom of Great Britain and Northern Ireland

Whitcomb, Mary Beth; California, USA

White, Joanna; New South Wales, Australia

Whitehouse, William; Indiana, USA

Whitfield‐Cargile, Canaan; Georgia, USA

Wielaender, Franziska; Germany

Wiggen, Kelly; Missouri, USA

Wilkins, Pamela; Illinois, USA

Willesen, Jakob; Denmark

Williams, Diane; California, USA

Wilson, Katherine; Virginia, USA

Windsor, Rebecca; Massachusetts, USA

Winston, Jenessa; Ohio, USA

Wise, Jessica; New South Wales, Australia

Wismer, Tina; New York, USA

Witonsky, Sharon; Virginia, USA

Wittek, Thomas; Austria

Wittenburg, Luke; California, USA

Witzel, Angela; Tennessee, USA

Wojtas, Justyna; Poland

Woldemeskel, Moges; Georgia, USA

Wolff, Ewan; Florida, USA

Wood, Carrie; Massachusetts, USA

Wood, Michael; Wisconsin, USA

Woods, Glynn; United Kingdom of Great Britain and Northern Ireland

Woods, J. Paul; Ontario, Canada

Woods, Katharine; Saskatchewan, Canada

Woolard, Kevin; California, USA

Woolums, Amelia; Mississippi, USA

Wright, Kathy; Ohio, USA

Wrzosek, Marcin; Poland

Wynn, Susan; Georgia, USA

Xenoulis, Panagiotis; Texas, USA

Yadav, Sampurna Nand; Assam, India

Yancey, Caroline; New York, USA

Yang, Mhan‐Pyo; Chungbuk, Korea (the Republic of )

Yang, Vicky; Massachusetts, USA

Yu, Jane; New South Wales, Australia

Yun, Taesik; Chungcheongbuk‐do, Korea (the Republic of )

Zentek, Juergen; Germany

Zidan, Natalia; Wisconsin, USA

Ziegler, Jessie; Arizona, USA

Zolgharni, Massoud; London, United Kingdom of Great Britain and Northern Ireland

Zur Linden, Alex; Ontario, Canada

